# Clinical and Biochemical Correlation of Intra-articular Platelet-Rich Plasma and Corticosteroid Using Serum Matrix Metalloproteinase 3 (MMP-3) Levels in Osteoarthritis of Knee

**DOI:** 10.7759/cureus.39625

**Published:** 2023-05-29

**Authors:** Vaneet Arora, Manmohan Sharma, Sandeep Bishnoi, Vakul Mahipal, Angad S Sandhu, Rajat Khanna, Tarun Aggarwal, Krishnadev S Yadav, Gautam Jain, Shubham M Sharma

**Affiliations:** 1 Orthopaedics, Teerthanker Mahaveer Medical College & Research Centre, Moradabad, IND; 2 Community Medicine, Jaipur National University Institute of Medical Sciences and Research, Jaipur, IND

**Keywords:** mmp-3, matrix metalloproteinase-3, methyl-prednisolone, corticosteroid, platelet-rich plasma, osteoarthritis knee

## Abstract

Introduction

Osteoarthritis (OA) in humans is an inevitable consequence of ageing and can now be effectively managed with advancements in knowledge and understanding of the disease. The major concern in a patient suffering from this disease is the functional impairment caused by the pain. The goals in the management of OA knee include symptom relief with preservation of joint function. Despite there being a number of studies on the effectiveness of PRP and CS for knee OA, most of them have focused on patient-reported functional outcomes only. Hence, we conducted this study to assess the potential and effectiveness of a single intra-articular injection of PRP and CS in the functional improvement of knee OA patients using the Western Ontario and McMaster Universities Osteoarthritis Index (WOMAC) and Visual Analogue Scale (VAS) and to establish the bio-modulatory effects of intra-articular PRP and CS in knee OA patients by estimating the serum matrix metalloproteinase-3 (MMP-3) levels.

Methodology

Patients attending the outpatient department with complaints of knee pain were screened. Standing anteroposterior and lateral radiographs of the knees were obtained. Patients with Kellgren and Lawrence (K-L) grades II and III were enrolled in our study. A total of 96 patients were included in the study after fulfilling the inclusion and exclusion criteria. Patients were divided into two groups (PRP and CS) by randomisation. There were 48 each in the PRP and CS groups, out of which nine were lost to follow-up, two from the PRP group and seven from the CS group. A total of 87 patients fulfilling the inclusion criteria were finally enrolled in the study and followed up for nine months after a single intra-articular injection. The biochemical assessment of serum levels of MMP-3 was done at baseline and in the ninth month. Accordingly, patients in the PRP group were injected with freshly prepared PRP (3 ml) within two hours of preparation, whereas those in the CS received 80 mg of methylprednisolone acetate. VAS and WOMAC were measured at baseline, and then in the first, third, sixth, and ninth month post-injection follow-ups. MMP-3 level was estimated before the injection and at the ninth-month post-injection follow-up. Data collected for both groups were analysed and compared with each other.

Conclusion

PRP is unquestionably a better option than CS in OA of the knee based on boosting functional activity, lowering stiffness, and reducing pain, all three of which are denoted by the WOMAC and VAS scores as the effect of PRP lasts longer than CS injections for the aforesaid issues. We could not find any significant change in levels of MMP3 post PRP and CS injections, which signifies that these two modalities do not have any effect in either preventing cartilage degeneration or promoting cartilage regeneration. Our findings have shown that PRP injections are safe, minimally invasive, and effective treatment modalities for OA knee.

## Introduction

Osteoarthritis (OA) in humans is an inevitable consequence of ageing and can now be effectively managed with advancements in knowledge and a better understanding of the disease. OA is multifactorial in origin and the knee is the most commonly involved joint. Any tissue in and around the joint can be affected, making it a pan-articular disease, but the hallmark of OA is articular cartilage degradation, which happens due to the inability of chondrocytes to maintain a homeostatic balance between the production of matrix and its breakdown, resulting in exposed bone beneath the cartilage [1]. The major concern in a patient suffering from this disease is the functional impairment caused by the pain [2].

The goals in the management of OA knee include symptom relief with preservation of joint function. Conservative management is usually the first line of management in early grades. There is a firm belief that to check the disease's progression in its early stages, it is essential to impart conservative management options to the patient in the form of lifestyle modification, weight reduction, quadriceps strengthening exercises, brace application whenever needed, along with oral medications like non-steroidal anti-inflammatory drugs (NSAIDs) and chondroprotective drugs [2-4]. Intra-articular injections of corticosteroid (CS), platelet-rich plasma (PRP), mesenchymal stem cells, and hyaluronic acid have been reported to provide symptomatic relief without systemic side effects. Still, the duration of their action, as well as local side effects, are questionable [4,5]. Among them, CS and PRP are the most economical and probably the most studied. CS disrupts inflammatory and immunological cascade at multiple levels by explicitly focusing on nuclear steroid receptors by reducing the capillary permeability, inhibiting inflammatory cell accumulation, phagocytosis, the production of neutrophil superoxide, metalloprotease, as well as the synthesis and production of several inflammatory mediators like prostaglandin [6,7]. PRP acts in more ways than just one, including chondrocyte production, bone remodelling, angiogenesis, anti-inflammatory effects, and cell differentiation [8,9]. Freshly prepared platelet plasma actively contributes to the healing process by releasing a variety of growth factors such as insulin-like growth factor, transforming growth factor b-1, and platelet-derived growth factor [10,11].

The matrix metalloproteinase family is gaining popularity as a promoter of cartilage extracellular matrix and basement membrane component degradation. Matrix metalloproteinase 3 (MMP-3) is naturally produced by chondrocytes and synovial cells and can not only degrade extracellular matrix but also activate other serine proteases [12-14].

Despite there being a plethora of studies on the effectiveness of PRP and CS for knee OA, the consensus is still not clear regarding the establishment of their bio-modulatory role. Most studies have focused on patient-reported functional outcomes only. Hence, we conducted this study to assess the potential and effectiveness of a single intra-articular injection of PRP and CS in the functional improvement of knee OA patients using the Western Ontario and McMaster Universities Osteoarthritis Index (WOMAC) and Visual Analogue Scale (VAS) to establish the bio-modulatory effects of intra-articular PRP and CS in knee OA patients by estimating the serum MMP-3 levels.

## Materials and methods

This was a single-centered prospective randomised study to compare the effects of intra-articular PRP and CS (methylprednisolone acetate) by accessing improvement in pain, stiffness, and physical function of patients suffering from OA knee and their effect on serum MMP-3 levels. The study was conducted from October 2020 to June 2022 in a tertiary care centre after getting approval from Teerthanker Mahaveer University's Institutional Ethical Committee (IEC) board (approval number: TMU/IEC/20-21/140). The study design, case record form (CRF), and consent form were approved by the College Research Committee and IEC.

Inclusion and exclusion criteria

The study included patients with knee OA diagnosed on the basis of the American College of Rheumatology (ACR) criteria. The grades of the knee OA were further classified according to the Kellgren and Lawrence (K-L) grading system and patients with K-L grades II and III were enrolled in our study [15].

The following patients were excluded: (i) patients who had any coronal and sagittal plane deformity >5 degrees, (ii) patients with any ligamentous instability around the knee, (iii) patients who had earlier undergone surgery for any lower limb pathology, (iv) Immunocompromised patients, (v) patients with any active or healed knee infection, any type of tumour, isolated patellofemoral arthritis, grade 4 patellofemoral arthritis, inflammatory arthropathies of knee, or any pathology of a unilateral or bilateral lower limb other than knee joint, (vi) patients having any bleeding abnormality, and (vii) patients with poorly controlled diabetes mellitus, hypertension, and any other chronic systemic illness.

Participants

Patients attending the outpatient department of orthopaedics with complaints of knee pain for a duration of less than two years with near normal range of motion and managed previously with conservative treatment with NSAIDs, opioids and braces were screened. Standing anteroposterior and lateral radiographs of knees were obtained which were then graded according to the K-L grading system. A total of 96 patients were enrolled in our study. Patients were divided into two groups (PRP and CS) by randomization technique, which was performed using an Internet platform called Research Randomizer v4.0 [16]. Nine were lost to follow-up; two from the PRP group and seven from the CS group. A total of 87 patients fulfilling the inclusion criteria were finally enrolled in the study and followed up for nine months after a single intra-articular injection.

Outcome measures

Assessment of pain was estimated based on VAS, which was measured on a 0 to 10 scale, with 0 being no pain and 10 denoting the worst possible pain. Functional assessment was done using the WOMAC, having a total score of 96 [17,18]. WOMAC score is a set of questionnaires that helps to estimate clinical improvement by assessing the osteoarthritic knee's pain, stiffness, and functional outcomes. The higher the score, the worse the function or symptoms. The biochemical assessment of serum levels of MMP-3 (ng/ml) was done at baseline and in the ninth month by commercially available enzyme-linked immunosorbent assay (ELISA) kits from KinesisDx (KRISHGEN BioSystems, Brea, California, United States). VAS and WOMAC were calculated at baseline, and then at the first, third, sixth and ninth months post injection.

Method of PRP preparation

After following all necessary aseptic precautions, 35-40 ml of blood was collected in 8.5 ml acid citrate dextrose (ACD) tubes from the antecubital vein of each patient. Then the blood was centrifuged at 3000 rpm using a soft spin for three minutes with the help of a Yorco centrifuge machine (York Scientific Industries Private Limited, Ghaziabad, Uttar Pradesh, India), available in our Department of Pathology's Blood Bank. To obtain a concentrate rich in platelets, the supernatant plasma which contains platelets was then transferred to another sterile tube of 10 ml (with no anticoagulant) and was again centrifuged at 4000 rpm using hard spin for a duration of 15 minutes. The lower one-third of this concentrate is PRP while the remaining upper two-thirds is PPP (platelet-poor plasma). Pellets of platelets are formed at the bottom of this tube. The upper two-thirds containing PPP was discarded and the remaining PRP was suspended in 3 ml of plasma by gently shaking the tube.

Method of injection administration

Patients in the PRP group were injected with freshly prepared PRP (3 ml) within two hours of preparation, whereas those in the CS group received 80 mg of methylprednisolone acetate. No local anaesthesia was used before injection. Under full aseptic precautions, PRP or CS was injected in the affected knee with a 22G needle using standard approaches (anterolateral approach) in the leg hanging from a table with 90⁰ flexion. After the injection, patients were told to perform an active range of motion at the knee. The patient was sent home after an observation of one hour. Patients were advised to limit their professional and occupational activities for a post-procedure period of 48-72 hours and thereafter were encouraged to do quadriceps strengthening exercises. They were allowed to take oral tramadol 50 mg twice daily, up to a maximum duration of 72 hours post-injection. The patients who reported having pain beyond the period of 72 hours were managed by extended use of an oral preparation of tramadol for a period of 10 days.

Statistical analysis

All analysis was performed using IBM SPSS Statistics for Windows, Version 20.0 (Released 2011; IBM Corp., Armonk, New York, United States). Mean and standard deviation were calculated for quantitative data and frequency. Percentages were calculated for qualitative data. The Chi-square test was used to find the association between categorical variables and to compare the mean, we used an independent t-test. The repeated measure ANOVA test was used to compare mean WOMAC and VAS scores at follow-up. The level of significance was considered as < 0.05 or 5%.

## Results

We enrolled a total of 96 patients, 48 in each group (PRP and CS), out of which nine were lost to follow-up, two from the PRP group and seven from the CS group. So, data from a total of 87 patients (46 in the PRP group and 41 patients in the CS group) were finally analysed. All demographic variables (age, gender and BMI) were comparable in both groups. The mean age in PRP and CS groups were 54.11 ± 9.56 and 54.54 ± 8.19, respectively. The gender distribution among the PRP and CS groups was not significant, with males in PRP and CS groups numbering 13 and 16, respectively, whereas females were 33 and 25, respectively. The baseline WOMAC, VAS, and MMP-3 were comparable in both groups; in PRP it was found to be 60.45 ± 11.648, 7.20 ± 0.859, and 48.884 ±5.668, respectively, whereas, in the CS group, it was found to be 57.28 ± 11.012,6 .83 ± 0.946, and 47.884 ±4.668, respectively. Baseline BMI was also comparable in both groups, which was 27.21 ± 3.68 in the PRP group and 27.05 ± 2.03 in the CS group as shown in Table 1.

**Table 1:** Demographic variables, BMI, WOMAC, VAS, MMP-3 at baseline CS: corticosteroid; PRP: platelet-rich plasma; WOMAC: Western Ontario and McMaster Universities Osteoarthritis Index; VAS: Visual Analogue Scale; MMP-3: matrix metalloproteinase 3; BMI: body mass index P >0.05, not significant

	Intervention groups		p-value
	PRP	CS	
Age (years)	54.11 ± 9.56	54.54 ± 8.19	0.824
Gender			0.288
Female, n (%)	33 (71.73)	25 (60.97)	
Male, n (%)	13 (28.26)	16 (39.02)	
WOMAC baseline	60.45 ± 11.648	57.28 ± 11.012	0.197
VAS baseline	7.20 ± 0.859	6.83 ± 0.946	0.052
MMP-3 baseline	48.884 ±5.668	47.884 ±4.668	0.375
BMI (kg/m^2^)	27.21 ± 3.68	27.05 ± 2.03	0.805

WOMAC

Comparison in PRP Group

The mean WOMAC score at baseline in the PRP group was 60.45 ± 11.648. When this was compared to mean WOMAC scores in the first (55.38), third (50.76), sixth (48.76), and ninth (52.39) months, we found that there was a continuous reduction in mean WOMAC score throughout the follow-up. However, the significant reduction was only in the third, sixth, and ninth months of follow-up as P-values came to be 0.001, 0.001, and 0.007, respectively, as shown in Table 2.

**Table 2:** Comparison of mean difference in WOMAC scores at baseline with the successive follow-ups in the PRP group WOMAC: Western Ontario and McMaster Universities Osteoarthritis Index; PRP: platelet-rich plasma P >0.05 not significant

Follow-Up	Mean Difference	P-Value	95% Confidence Interval	
			Lower Bound	Upper Bound
At 1 Month	5.07	0.212	-1.57	12.31
At 3 Months	9.69	0.001	3.28	17.15
At 6 Months	11.69	0.001	5.28	19.15
At 9 Months	8.06	0.007	1.65	15.52

Comparison in CS Group

The mean WOMAC score at baseline in the CS group was 57.28. When this was compared to mean WOMAC scores at the successive follow-ups, that is at first (51.54), third (52.22), sixth (55.51), and ninth (59.85) months, it was found that there was a continuous decrease in WOMAC score till six months of follow-up but at the ninth month of follow-up, the WOMAC score increased beyond the baseline score. Though there was a decrease in WOMAC score till the sixth month, the decrease was insignificant on comparing these follow-up scores to baseline scores as shown in Table 3, denoting that CS helps in insignificant improvement in functional score.

**Table 3:** Comparison of mean difference in WOMAC scores at baseline with the successive follow-ups in the CS group WOMAC: Western Ontario and McMaster Universities Osteoarthritis Index; CS: corticosteroid P >0.05 not significant

Follow Up	Mean Difference	P-Value	95% Confidence Interval	
			Lower Bound	Upper Bound
At 1 Month	5.74	0.168	-1.29	12.60
At 3 Months	5.06	0.209	-1.55	12.33
At 6 Months	1.77	0.921	-4.85	9.04
At 9 Months	-2.57	0.900	-9.19	4.70

Comparison Between Both Groups

When the mean of WOMAC score was analysed in between the groups (PRP and CS) at baseline and the first, third, sixth, and ninth-month follow-ups, we found that there was a significant reduction of mean WOMAC score at the sixth and ninth months in PRP when compared to that of CS group, whereas there was no significant reduction in mean at other follow-ups as depicted in Table 4.

**Table 4:** Comparison of mean WOMAC scores between groups CS: corticosteroid; PRP: platelet-rich plasma; WOMAC: Western Ontario and McMaster Universities Osteoarthritis Index; SD; standard deviation P >0.05 not significant

Variable	Group	Number of participants	Mean	SD	P-value
Before Injection (Baseline)	CS	41	57.28	11.02	0.171
	PRP	46	60.45	11.65	
At first month	CS	41	51.54	11.58	0.156
	PRP	46	55.38	12.15	
At third month	CS	41	52.22	11.87	0.563
	PRP	46	50.76	11.56	
At sixth month	CS	41	55.51	11.60	0.009
	PRP	46	48.76	12.05	
At ninth month	CS	41	59.85	11.00	0.005
	PRP	46	52.39	13.03	

VAS

Comparison in PRP Group

The mean VAS score at baseline in the PRP group was 7.20. When this was compared to mean VAS scores at the successive follow-ups, that is at first (6.72), third (5.52), sixth (5.28), and ninth (5.91) months, we found that VAS score continuously reduced throughout the follow-ups, and the reduction was significant at every follow-up month as P-values came to be 0.004, 0.001, 0.001, and 0.001, respectively, as shown in Table 5, denoting that effect of PRP in decreasing the pain lasted till ninth months or more.

**Table 5:** Comparison of mean difference in VAS scores at baseline with the successive follow-ups in the PRP group VAS: Visual Analogue Scale; PRP: platelet-rich plasma P >0.05 not significant

Follow-Ups	Mean Difference	P-Value	95% Confidence Interval	
			Lower Bound	Upper Bound
At 1 Month	0.48	0.004	0.16	1.24
At 3 Months	1.68	0.001	1.13	2.21
At 6 Months	1.92	0.001	1.37	2.45
At 9 Months	1.29	0.001	0.74	1.82

Comparison in CS Group

The mean VAS score at baseline in the CS group was 6.83. When this was compared to mean VAS scores at the successive follow-up, that is at first (6.72), third (5.71), sixth (6.27) and ninth (7.24) months, it was found VAS score continues to decrease till six months of follow-up but at the ninth month of follow-up, the VAS score increased beyond the baseline score. Moreover, there was a significant decrease in VAS score till the third month only, denoting that the role of CS in decreasing pain weans off after a period of three months, as shown in Table 6.

**Table 6:** Comparison of mean difference in VAS scores at baseline with the successive follow-ups in the CS group VAS: Visual Analogue Scale; CS: corticosteroid P >0.05 not significant

Follow-Ups	Mean Difference	P-Value	95% Confidence Interval	
			Lower Bound	Upper Bound
At 1 Month	0.15	0.001	0.60	1.98
At 3 Months	1.12	0.001	0.43	1.81
At 6 Months	0.56	0.171	-0.13	1.25
At 9 Months	-0.41	0.466	-1.11	0.28

Comparison Between Both Groups

When the mean of the VAS score was analysed in between the groups (PRP and CS) at baseline and the first, third, sixth, and ninth-month follow-ups, we found that there was a significant reduction of mean VAS score in the sixth and ninth months, whereas there was no significant reduction in mean VAS scores in PRP group when compared to CS group in the sixth and ninth-month follow-ups as depicted in Table 7; this reveals that effect of PRP is long-lasting.

**Table 7:** Comparison of mean VAS scores between PRP and CS groups. CS: corticosteroid; PRP: platelet-rich plasma; VAS: Visual Analogue Scale; SD: standard deviation P >0.05 not significant

Variable	Group	Number of Participants	Mean	SD	P-value
Before injection	CS	41	6.83	0.95	0.062
	PRP	46	7.20	0.86	
At first month	CS	41	6.68	1.14	0.465
	PRP	46	6.72	0.86	
At third month	CS	41	5.71	1.17	0.387
	PRP	46	5.52	0.81	
At sixth month	CS	41	6.27	1.28	0.001
	PRP	46	5.28	0.96	
At ninth month	CS	41	7.24	1.11	0.001
	PRP	46	5.91	1.17	

Serum MMP-3

In the comparison of the mean of MMP-3 in K-L grades 2 and 3 in CS Group and PRP Group, it was found that there was an insignificant change in serum levels of MMP-3 in both groups, as shown in Table 8. This shows that neither PRP nor CS has any role in decreasing the levels of MMP-3.

**Table 8:** Comparison of mean MMP-3 levels at baseline with mean MMP-3 levels at the ninth-month follow-up CS: corticosteroid; PRP: platelet-rich plasma; MMP-3: matrix metalloproteinase 3 P >0.05 not significant

Biomarker	Group	Baseline Mean	Mean at 9-Month Follow-up	P-Value
MMP-3	PRP	48.8843 (±5.6683)	47.7378 (±3.1984)	p = 0.2353
	CS	47.8843 (±4.6683)	46.7378 (±2.1984)	p = 0.1587

## Discussion

Knee OA is the most prevalent degenerative joint disease in the world. Reducing pain, enhancing function, enhancing the quality of life, and slowing the development of the disease are the key therapeutic objectives. However, there are presently no treatments that can stop the growth of knee OA. Surgery can help alleviate pain and improve joint mobility and function, but it is expensive and has a risk of morbidity. Analgesics and NSAIDs have only marginal effectiveness. Intra-articular injections of hyaluronic acid and CS offer temporary relief from OA discomfort. Recent trials using a placebo control group have demonstrated that intra-articular PRP injections can reduce pain while enhancing knee function and quality of life [1].

In the present study, we enrolled 96 patients of OA knee with K-L grades 2 and 3 by randomizing them into two groups of 48 patients each, the first being PRP and the second was CS to compare their effectiveness. Out of these, nine were lost to follow-up, so data from a total of 87 patients (46 in the PRP group and 41 patients in the CS group) were finally analysed. Age, gender distribution, BMI, and baseline WOMAC and VAS scores were comparable in both groups.

We administered a single intra-articular injection (PRP or CS) in the knee after taking the baseline WOMAC and VAS scores and followed up on these scores in the first, third, sixth, and ninth months to evaluate the functional outcome. A baseline MMP-3 level was noted for every patient who was followed up at the ninth month post injection. We found that a single injection of intra-articular PRP significantly reduces the WOMAC score at the third, sixth, and ninth-month follow-ups when compared to pre-injection WOMAC score, which depicts that PRP has an effect lasting for around nine months or more in the improvement of joint stiffness, pain, and functional outcome (all collectively depicted by WOMAC score). We compared the mean VAS score at baseline in the PRP group to mean VAS scores at every follow-up. We found a continuous significant reduction in mean VAS score throughout the follow-ups, further potentiating our results. On the other hand, in the CS group, we compared the mean WOMAC score at baseline with the mean at every follow-up and found that there was no significant improvement at any follow-up month in the WOMAC score rather, at the ninth month of follow-up, the WOMAC score worsened when compared to the baseline. Moving forward, we compared the pre-injection mean VAS score to mean VAS at every follow-up and found a significant reduction in VAS until the first three months only, after which there was no significant reduction of pain; instead, the pain worsened in the ninth month when compared to that of baseline. Hence, we can conclude that the action of CS has a definite role in decreasing pain, but the period of action is short, and further, we did not find any role of CS in improving functional outcomes. PRP has a definite role in improving knee function, which is significant and longer lasting as proven by our study and other recent literature.

A study by Arjun et al. compared the effect of PRP with that of CS in OA knee; a total of 60 subjects was randomised between both groups [19]. The outcome assessment tools used by them were similar to that of our study, that is, WOMAC and VAS scores, and followed up for 24 weeks. They concluded that the PRP has a better effect in decreasing the WOMAC and VAS scores when compared to that of CS, which is in harmony with our study.

A study by Bharath and Uidesh sought to contrast the intra-articular injection techniques of PRP vs CS and evaluate the knee's functional result [20]. A total of 100 volunteers were signed up for the study and distributed equally between the groups. The WOMAC and VAS scores are adopted to evaluate the functional result of the knee. Scores were formerly collected in the sixth week, third month, and sixth month. The findings led the researchers to conclude that PRP might be considered an attractive therapy modality for treating knee OA because it has shown promising results in long-term (up to six months) instances. The study depicted that the baseline characteristics (mean age, gender, baseline WOMAC and VAS, and BMI) were comparable in both groups

In their comprehensive review and meta-analysis, McLarnon et al. included eight trials comprising 648 patients, of whom 443 (68%) were female and had a mean BMI of 28.4 and an age of 59 years. The authors came to the same conclusion as our trial that intra-articular PRP was more effective than CS at reducing OA symptoms at the third, sixth, ninth, and 12 months [21].

PRP is supposed to work at the biological level and helps in cartilage regeneration and decrease inflammation by virtue of growth factors present in it. To control OA knee, the property of the substance should be such that it should help in the regeneration of damaged cartilage and prevent further degradation. To assess cartilage degeneration, serum MMP-3 level is a good biomarker; therefore, to assess cartilage degeneration, we assessed the serum MMP-3 level before the injection and at the ninth-month follow-up post injection.

We did not find any significant reduction in mean MMP-3 levels when the baseline mean MMP-3 levels were compared to the level of mean MMP-3 at the ninth-month follow-up in any of the groups.

In a single-center, prospective clinical research, Lacko et al. enrolled 40 patients with unilateral primary knee OA [22]. For each patient, three intra-articular PRP injections were given, each one spaced by a week. Clinical and analytical evaluations were carried out prior to the first PRP injection (baseline) and three months following the third PRP treatment (three-month follow-up). The VAS for pain was used to evaluate the affected knee joint's level of pain. The WOMAC was used to assess changes in clinical status. They measured the concentrations of 19 biomarkers in the serum of the patients under study and came to the conclusion that PRP injection had no significant impact on cytokine levels in plasma.

It has already been established that PRP is used worldwide as a disease-modifying agent, and its efficacy in improving functional outcomes and quality of life has already been proved. Our results support this effect of PRP in patients of OA knee, but we have failed to prove the effect of PRP in the reduction of cartilage degeneration by analyzing serum MMP-3 levels.

The limitations of our study were that it was a single-centre study with a low sample size, so we could not analyze the wide variation in the target population. The follow-up in our study was short, that is only nine months, so we could not assess the effect of PRP beyond nine months. We did not enrol any controls in our study, and due to the lack of imaging to see cartilage volume change, the outcome score was subjective, and we only used a single marker.

## Conclusions

Finally, we want to conclude that PRP is unquestionably a better option than CS in OA of the knee based on boosting functional activity, lowering stiffness, and reducing pain, all three of which are denoted by the WOMAC and VAS scores as the effect of PRP lasts longer than CS injections for aforesaid issues. We could not find any significant change in levels of MMP3 post PRP and CS injection, which signifies that these two modalities do not have any effect in either preventing cartilage degeneration or promoting cartilage regeneration. Our findings have shown that PRP injections are safe, minimally invasive, and effective treatment modalities for OA knee.
